# Supplementary material for: An open-source framework for large-scale, flexible evaluation of biomedical text mining systems
Source: J Biomed Discov Collab. 2008 Jan 29;3:1. doi: 10.1186/1747-5333-3-1 (PMC2276192; doi:10.1186/1747-5333-3-1)
Supplement: Additional file 1 — Evaluation platform source code. The additional file contains the software evaluation framework discussed in this paper. The most current release can be downloaded from SourceForge.net [1]. See the accompanying README file for instructions on installation and use. Also included in the distribution is a collection of UIMA wrappers for some commonly used BioNLP tools and annotated corpora. [file 1747-5333-3-1-S1.zip › bionlp_uima_repository-1.0.1/docs/api/src-html/edu/uchsc/ccp/util/nlp/annotation/comparison/SharedEndSpanComparator.html]

```
001    package edu.uchsc.ccp.util.nlp.annotation.comparison;
002    
003    import java.util.List;
004    
005    import edu.uchsc.ccp.util.nlp.annotation.Span;
006    
007    /**
008     * 
009     * For "shared end" comparison, the spans are required to have the same end index in order to return 0. If they do not share the same span end, then
010     * if the span start for span1 is prior to the span start for span2, then -1 is returned, 1 otherwise.
011     * 
012     * @author williamb
013     * 
014     */
015    public class SharedEndSpanComparator extends SpanComparator {
016    
017        /**
018         * Compare two spans to see if they share the same end.
019         */
020        @Override
021        public int compare(Span span1, Span span2) {
022            return sharesSpanEnd(span1, span2);
023        }
024    
025        /**
026         * Compare two lists of spans using the shared-end match criteria.
027         * @param spanList1
028         * @param spanList2
029         * @return
030         */
031        @Override
032        public int compare(List<Span> spanList1, List<Span> spanList2) {
033            return sharesSpanEnd(spanList1, spanList2);
034        }
035    
036    }
```
